# The Use of Nosophen and Antinosin in Purulent Disease of the Middle Ear1Read before the Buffalo Academy of Medicine June 7, 1898.

**Published:** 1898-12

**Authors:** Frederick H. Millener

**Affiliations:** Buffalo, N. Y. 5 North Pearl Street


### THE USE OF NOSOPHEN AND ANTINOSIN IN PURULENT DISEASE OF THE MIDDLE EAR.[1]

By FREDERICK H. MILLENER, M. D., Buffalo, N. Y.

WITHIN the last few months, after having suffered much disappointment incident upon the unsuccessful treatment of otitis media purulenta, I was induced to try the use of nosophen, and its sodium salt antinosin, a new antiseptic. These substances were first described by Loeb and Classen. Nosophen consists of a very light yellowish-grey, odorless, tasteless powder containing 61 7-10 per cent. of iodine in combination. It does not readily decompose and may be heated to 220° centigrade. It is insoluble in water and acids, slightly soluble in alcohol, glacial acetic acid, ether and chloroform, but readily soluble in alkalies, with the last of which it forms salts, from which it is freed at low temperature by carbon-dioxide. By virtue of its hydroxyl groups, nosophen is an acid readily soluble in alkalies with the replacement of the H. atoms of both hydroxyl groups by a metal. The sodium salt thus originated is readily isolated and is known as antinosin, a sodium salt of tetra-iodo-phenol-phtalien. It is a dark blue amorphous powder which is readily soluble in water and alcohol. Contrary to nosophen it is changed by continued exposure to the carbonic acid gas of the air, thereby being transformed into nosophen and carbonate of soda. It is odorless or nearly so, nontoxic and nonirritant.

These two preparations, nosophen and antinosin, according to Doctors Loeb and Lieven, are in direct opposition to those iodine compounds which readily liberate iodine. The atoms of iodine are so firmly combined that liberation of iodine is excluded. Nosophen can be boiled for hours in caustic soda, and this property is of great importance, since it explains the nontoxic action of nosophen and its salts within the organism.

Unlike iodoform, which acts by virtue of its decomposition within the body itself, with the liberation of free iodine, nosophen and antinosin liberate no iodine. On the contrary nosophen manifests its action as a nondecomposed molecule. The researches of Lieven, Seifert, Binz and Zuntz on both man and animals show that nosophen or antinosin when introduced into the organism by absorption, subcutaneously or otherwise, reappears as nosophen in the excreta. If the toxic phenomena which are known as iodin

1. Read before the Buffalo Academy of Medicine June 7, 1898.

intoxications are referable to the liberation of iodine, then nosophen and antinosin must be nontoxic.

Nosophen and antinosin have been administered to dogs without giving rise to the least untoward symptoms. The passage of the drug through the digestive tract causes no irritation, no trace of free iodine is found in the urine; they are therefore nontoxic. The possible media through which the antiseptic properties of nosophen and antinosin are exerted are the iodo-phenol-phtalien albuminate, *i. e.*, soluble iodine compounds.

The mode of procedure and the effect of these new drugs is well illustrated in the following cases:

CASE I.—M. L., a girl of 15. Since scarlet fever, at the age of 8, a purulent discharge from both ears has been more or less constant. First syringing the ears with water as warm as she could bear, as much antinosine solution (strength 2½ per cent.) as would fill the ears was dropped in and allowed to remain five minutes. This was done twice a day for three days. On the fourth day the discharge was much lessened, but a severe cold was then unfortunately contracted and as a result an increase in the discharge took place. A 3 per cent. solution was then used, and after removal of the same, nosophen was applied as a powder by means of a powder blower, merely covering the mucous membrane and tympanum, not packing or plugging the external auditory canal in any way. On instilling the 3 per cent. solution the patient complained of a dizziness which continued for several minutes. This treatment, regularly carried out every other day for fourteen days, caused the cessation of the discharge and now, nearly a year later, there has been no recurrence. In addition, I may add, the nasopharynx was regularly washed out with an alkaline wash and kept free from any accumulation of mucus.

CASE II.—M. E., 18 years of age. Seen August 6, 1897. Discharge from both ears for thirteen years, which had been treated with innumerable drugs, but with little success. Hearing distance, right ear, ten to thirty feet. Hearing distance, left ear, twelve to thirty feet.

Before commencing treatment I removed a portion of the hypertrophied inferior turbinated bone on each side, after which potassium permanganate solution was given her to be used as directed, but with little result. Antinosin and nosophen were then used in the manner above described, with the result that in fourteen days the discharge ceased and as yet has not returned.

CASE III.—J. B., child, aged 7. Purulent discharge since scarlet fever, two years ago. Large eczematous excoriations upon the tragus, antitragus and lobes of the ear. Treated with yellow oxide of mercury and antinosin and nosophen used as before. At the end of three weeks the discharge ceased, but the treatment was ordered continued one week longer. No recurrence.

CASE V.—A. G., girl, aged 10. History of la grippe. Examination revealed presence of adenoid vegetations and enlarged tonsils; also a profuse discharge from the ear. Upon the removal of these growths the usual treatment followed. The child's hearing at once improved. No recurrence of the discharge.

The duration of treatment with permanganate of potash depended largely upon the frequency with which it was used. If used regularly the discharge would stop only to continue again when the remedy was discontinued. Occasionally an ear would cease discharging and as far as I know not commence again, but this was the exception and not the rule.

Nosophen and antinosin have now been used by me in thirty-six cases. Twenty-six of these were acute otitis media purulenta and had adenoid vegetations and they made a rapid recovery in twenty to thirty days; sixteen were chronic otitis media purulenta and several of these had growths in the throat or vault of the pharynx (adenoid vegetations) and enlarged tonsils, which were removed. This is mentioned because I wish to point out the importance of removing these growths if present, as no amount of medicine will permanently cure an ear which is discharging from these causes. Five of the thirty-six were very much improved, and I consider one of the reasons the results in these cases are not better is because they did not follow the directions carefully. Five I have been unable to hear from, owing to their having given wrong addresses.

In cases where the discharge is due to granulation tissue forming or to caries of the ossicles, the diseased portions of the bone must be removed (and if due to granulation tissue, that also must be removed before the medicine is applied). In cases of acute otitis media purulenta, whether due to adenoid vegetations or as a sequela of the infectious fevers, I find the best results are obtained by the use of the 3 per cent. solution.

The drugs which had been most in favor with me previous to the use of nosophen and antinosin, were permanganate of potassium, boracic acid, sulphate of zinc, corrosive sublimate, tannic acid, peroxide of hydrogen and others, but the remedies from which the best results were secured were nosophen and antinosin. They invariably followed the use of the former drugs with more or less happy results. Dr. E. P. Gleason, of Philadelphia, states that when nosophen is properly applied, after a thorough cleansing with antinosin, it is capable of acting as a sedative antiseptic powder to a marked degree. I have noticed that it possesses one disagreeable action, which is, that after using a 3 per cent. solution, the patient is affected by a marked dizziness, and this dizziness is due to the antinosin and not to the fact that it is a fluid put in the ear with too much force, for it matters not how gently you inject it the dizziness occurs. This need not alarm the physician as the symptom is quickly over and the patient rapidly improves.

I do not wish to be understood that this is an exclusive remedy for all discharges from the middle ear, but simply to state that in my opinion it has demonstrated its superiority over other drugs in general use for this condition, if properly used.

5 North Pearl Street.

